# *In vitro *and *in vivo *pre-clinical analysis of a F(ab')_2 _fragment of panitumumab for molecular imaging and therapy of HER1-positive cancers

**DOI:** 10.1186/2191-219X-1-1

**Published:** 2011-06-07

**Authors:** Karen J Wong, Kwamena E Baidoo, Tapan K Nayak, Kayhan Garmestani, Martin W Brechbiel, Diane E Milenic

**Affiliations:** 1 Molecular Imaging Program, Center for Cancer Research, National Cancer Institute, National Institutes of Health, Bethesda MD 20892, USA; 2 Radioimmune and Inorganic Chemistry Section, Radiation Oncology Branch, Center for Cancer Research, National Cancer Institute, National Institutes of Health, 10 Center Drive MSC-1002, Bethesda MD 20892, USA

## Abstract

**Background:**

The objective of this study was to characterize the *in vitro *and *in vivo *properties of the F(ab')_2 _fragment of panitumumab and to investigate its potential for imaging and radioimmunotherapy.

**Methods:**

The panitumumab F(ab')_2 _was generated by enzymatic pepsin digestion. After the integrity and immunoreactivity of the F(ab')_2 _was evaluated, the fragment was radiolabeled. *In vivo *studies included direct quantitation of tumor targeting and normal organ distribution of the radiolabeled panitumumab F(ab')_2 _as well as planar γ-scintigraphy and PET imaging.

**Results:**

The panitumumab F(ab')_2 _was successfully produced by peptic digest. The F(ab')_2 _was modified with the CHX-A"-DTPA chelate and efficiently radiolabeled with either ^111^In or ^86^Y. *In vivo *tumor targeting was achieved with acceptable uptake of radioactivity in the normal organs. The tumor targeting was validated by both imaging modalities with good visualization of the tumor at 24 h.

**Conclusions:**

The panitumumab F(ab')_2 _fragment is a promising candidate for imaging of HER1-positive cancers.

## Background

Monoclonal antibodies (mAb) have been used in medicine for nearly three decades for purposes including imaging and therapy due to their selectivity for specific targets [1]. While intact monoclonal antibody molecules are still most commonly used, they may not necessarily be the most efficient or desired molecular form depending on the application. Because of their relatively large size (approximately 150 kD), intact mAbs tend to have unfavorable imaging kinetics, relatively poor tumor penetration, and present with the potential for eliciting host antibody responses [2-7]. The solution to these myriad obstacles has been to reduce the size of intact antibodies to smaller forms or fragments, achieved either through enzymatic cleavage or by genetic engineering. The latter strategy requires a serious commitment of time and resources while enzymatic methods for generating monovalent or bivalent fragments of a mAb is somewhat facile with a lesser investment incurred.

The bivalent F(ab')_2 _antibody fragment can be generated by cleaving the antibody on the carbonyl side of cysteinyl residues, below the disulfide bonds with pepsin [8]. This results in an Fc and an F(ab')_2 _fragment [9]. The removal of the Fc portion during digestion also removes the potential of binding with Fc receptors thus reducing non-specific interactions [10]. The average molecular weight of the F(ab')_2 _fragment is approximately 110 kD.

Radiolabeled mAbs are utilized in applications that include monitoring of tumor response to therapy, detection of metastatic lesions, dosimetric calculations, and therapy [10,11]. Again, mAb fragments may be preferable for several reasons. The removal of the Fc segment could reduce the non-specific distribution *in vivo *of the mAb via the Fc receptors found on normal cells. F(ab')_2 _fragments differ in their pharmacokinetic characteristics compared to intact antibodies resulting in distinct blood clearance and tumor localization patterns, clearing faster from the circulation than intact antibody while demonstrating better penetration into tumor sites [7,12-19]. The rapid clearance from the blood compartment by F(ab')_2 _results in a higher signal-to-noise ratio at earlier time points. A more favorable scenario for the imaging of patients is thus provided.

The smaller size and rapid clearance of antibody fragments such as F(ab')_2 _should also lower their immunogenicity potential, reducing the risk of patients developing a humoral response against the antibody fragment, and potentially permitting repeated treatment of patients [20]. The ability to administer multiple doses of mAb for either therapy or imaging has not been a trivial consideration in the management of cancer patients.

Panitumumab (ABX-EGF, Vectibix™, Amgen, Thousand Oaks, CA, USA) is a fully human IgG_2 _mAb that binds to the epidermal growth factor receptor (EGFR) with high affinity [21]. Panitumumab gained FDA-approval in 2006 for the treatment of patients with EGFR expressing metastatic colorectal carcinoma with disease progression while on or following fluoropyrimidine-, oxaliplatin-, or irinotecan-containing chemotherapy regimens [22]. Panitumumab has been well tolerated in clinical trials and as a result, close observation of patients has not been required nor has pre-medication with antihistamines [23]. The intact antibody has been shown to be successfully radiolabeled with ^111^In in high yields and has demonstrated excellent tumor targeting with low normal tissue uptake [24,25]. Panitumumab has also been successfully used for positron-emission tomography (PET) imaging using ^86^Y [26,27].

Extensive studies have been performed on the intact panitumumab; to date, there are no reports utilizing a fragment of panitumumab for either imaging or therapeutic applications. This paper represents the first *in vitro *and *in vivo *characterization of panitumumab F(ab')_2 _fragment with an emphasis on its evaluation towards both imaging and therapeutic applications.

## Materials and methods

### Preparation of F(ab')_2 _fragments

Panitumumab (Amgen) was dialyzed against 0.1 M sodium acetate, pH 4, using a 10 kD molecular-weight cut-off (MWCO) dialysis cassette (Pierce, Rockford, IL, USA). The solution was changed three times a day over the course of 4 days. The total quantity of recovered protein was determined by absorbance at 280 nm. To determine the optimal digestion time, panitumumab (250 μg) was digested at 37°C for 1, 2, 4, 6, 8 h and overnight with 2% (2.5 μg) pepsin (Sigma, St.Louis, MO, USA). Enzymatic activity was halted with the addition of 25 μL of 0.15 M carbonate solution. The digests were then analyzed by polyacrylamide gel electrophoresis (sodium dodecyl sulfate (SDS)-PAGE) using a 4-20% tris-glycine gel (Invitrogen, Carlsbad, CA, USA); samples without pepsin kept at 4°C and 37°C were included for comparison. Samples (25 μg) were applied to the gels both with and without β-mercaptoethanol in the sample buffer.

Larger preparations of panitumumab F(ab')_2 _were then generated in two stages. Conditions of the peptic digest were confirmed by producing F(ab')_2 _fragments using 100 mg of panitumumab. Following an overnight digestion with 2% pepsin at pH 4 in 0.1 M sodium acetate, the preparation was analyzed by size-exclusion high-performance liquid chromatography (SE-HPLC) and then dialyzed against phosphate-buffered saline (PBS) over the course of 4 days, three changes per day. The final protein concentration of the panitumumab F(ab')_2 _was determined by the Lowry method using a BSA standard [28] and the product was analyzed by SDS-PAGE. Upon completion of the analysis, F(ab')_2 _fragments were then prepared from 1 g of panitumumab. In this situation, a tangential flow filtration system (Millipore, Billerica, MA, USA) was used to exchange the preparation into PBS.

F(ab')_2 _fragments of trastuzumab and HuM195, an anti-CD33 mAb (a gift from Dr. McDevitt, Memorial Sloan Kettering Cancer Center) were also prepared using the conditions described for panitumumab.

### Conjugation and radiolabeling of panitumumab F(ab')_2_

Panitumumab F(ab')_2 _was conjugated with the bifunctional acyclic trans-cyclohexyl-diethylenetriamine-pentaacidic acid (CHX-A"-DTPA) chelate by a modification of established methods using fivefold, tenfold, and 20-fold molar excess of chelate to panitumumab F(ab')_2 _[29,30]. The final concentration was determined by the Lowry method. The average number of CHX-A"-DTPA molecules linked to the panitumumab F(ab')_2 _was determined using a spectrophotometric assay based on the titration of yttrium-Arsenazo(III) complex [31]. The HuM195 F(ab')_2 _fragment was conjugated with a tenfold molar excess of CHX-A"-DTPA.

Radiolabeling of the panitumumab F(ab')_2_-CHX-A"-DTPA with either ^111^In or ^86^Y was performed as previously described [32]. Radio-iodination of panitumumab (50 μg) with Na^125^I (0.5-1 mCi; PerkinElmer, Shelton, CT, USA) was performed using Iodo-Gen (Pierce Chemical, Rockford, IL, USA) [29,33].

### Cell culture

LS-174T cells, kindly provided by Dr. J. Greiner, NCI, were grown in Dulbecco's Modified Eagle's Medium, supplemented with 10% FetalPlex (Gemini Bio-Products, Woodland, CA, USA), 1 mM L-glutamine and 1 mM non-essential amino acids (NEAA). All media and supplements were obtained from Quality Biological (Gaithersburg, MD, USA) or Lonza (Walkersville, MD, USA). The cells were maintained in a 5% CO_2 _and 95% air-humidified incubator.

### Radioimmunoassays

The immunoreactivity of the panitumumab F(ab')_2 _was evaluated in a competition radioimmunoassay using purified human epidermal growth factor receptor (hEGFR; Sigma-Aldrich). Fifty nanograms of hEGFR in 100 μL of PBS containing Mg^+2 ^and Ca^+2 ^was added to each well of a 96-well plate. Following an overnight incubation at 4°C, the solution was removed and 1% bovine serum albumin (BSA) in phosphate-buffered saline (BSA/PBS,150 μL) was added to each well for 1 h at ambient temperature. The solution was removed and serial dilutions (1,000-17 ng) of the panitumumab F(ab')_2 _(50 μL) were added to the wells in triplicate; one set of wells received only BSA/PBS. After adding ^125^I-panitumumab (28 nCi in 50 μL) to each well, the plates were incubated at 37°C. At the end of the 4 h incubation, the solution was removed and the wells were washed three times with BSA/PBS. The radioactivity was removed from the wells by adding 0.2 N NaOH and adsorbing the liquid with cotton filters. The filters were then placed in 12 × 75 mm polypropylene tubes and counted in a γ-counter (WizardOne, Perkin Elmer, Shelton, CT, USA). The percent inhibition was calculated using the control (no competitor) and plotted. The panitumumab fragment was compared to intact panitumumab. Trastuzumab F(ab')_2 _was used as a negative control. All values were corrected for on a nanomolar basis.

The immunoreactivity of the ^111^In-panitumumab F(ab')_2 _was assessed in a radioimmunoassay as detailed previously using purified hEGFR [29,34]. Serial dilutions of ^111^In-CHX-A"-panitumumab F(ab')_2 _(approximately 200,000 to 12,500 cpm in 50 μL of BSA/PBS) were added to the wells of a 96-well plate coated with 100 ng of hEGFR in duplicate. Following a 4 h incubation at 37°C, the wells were washed, the radioactivity removed and counted in a γ-scintillation counter. The percentage binding was calculated for each dilution and averaged. The specificity of the radiolabeled panitumumab F(ab')_2 _was confirmed by adding 10 μg of unlabeled panitumumab to one set of wells.

### *In vivo *studies

#### Quantitation of tumor targeting

All animal care and experimental protocols were approved by the National Cancer Institute Animal Care and Use Committee. The *in vivo *behavior of the radioimmunoconjugate (RIC) F(ab')_2 _was assessed using LS-174T tumor bearing athymic mice (Charles River Laboratories, Wilmington, MA, USA). Four- to six-week old female mice received either subcutaneous (s.c.) injections in the flank with 2 × 10^6 ^cells in 0.2 mL of media containing 20% Matrigel ™(Becton Dickinson, Bedford, MA, USA) or intraperitoneal (i.p.) injections of 1 × 10^8 ^cells in 1 mL of media. Animals bearing s.c. tumors were used for the *in vivo *studies when the tumor diameter measured 0.4-0.6 cm. Mice with i.p. xenografts were utilized in studies at 4-5 days post-tumor implantation.

Tumor targeting was quantitated by injecting mice (*n *= 5 per time point) intravenously (i.v.) via tail vein or i.p. with ^111^In-CHX-A"-panitumumab F(ab')_2 _(approximately 7.5 μCi). The mice were euthanized at 24, 48, 72, 96, and 168 h. The blood, tumor, and major organs were collected, wet-weighed, and counted in a γ-scintillation counter. The percentage of injected dose per gram (%ID/g) was determined for each tissue. The averages and standard deviations are also presented.

#### Blood pharmacokinetics

Blood pharmacokinetics were performed with non-tumor bearing (*n *= 5) and mice (*n *= 5) bearing LS-174T (s.c. or i.p.) xenografts. ^111^In-CHX-A"-panitumumab F(ab')_2 _(approximately 7.5 μCi in 200 μL PBS) was administered by i.v. or i.p. injection, blood samples (10 μL) were collected in heparinized capillary tubes and the radioactivity measured in a γ-scintillation counter. The percent injected dose per milliliter (%ID/mL) was calculated for each of the samples and the average with the standard deviation plotted for each time point.

#### Imaging

γ-Scintigraphy was performed with tumor bearing mice to further validate the ^111^In-CHX-A"-panitumumab F(ab')_2 _tumor targeting. Imaging studies were performed with s.c. tumor bearing mice (*n *= 4) given i.v. injections of ^111 ^In-CHX-A"-panitumumab F(ab')_2 _(approximately 100 μCi in 0.2 mL PBS). The mice were chemically restrained with 1.5% isoflurane (Abbott Laboratories, North Chicago, IL, USA) delivered in O_2_, using a model 100 vaporizer (SurgiVet, Waukesha, WI, USA) at a flow rate of approximately 1.0 L/min. Images (100,000 counts) were acquired at 24, 48, 72, and 96 h using MONICA (Mobile Nuclear Imaging Camera, NIH, Bethesda, MD, USA) [35]. Images were analyzed using NucLear Mac software (Scientific Imaging, Inc., Crested Butte, CO, USA).

PET imaging study was performed using the Advanced Technology Laboratory Animal Scanner (ATLAS, National Institutes of Health, Bethesda, MD, USA) as previously described [32-34]. Whole-body imaging studies (six bed positions, total acquisition time of 1 h per mouse) were carried out on mice anesthetized with 1.5% isoflurane on a temperature-controlled bed as described previously [27]. In brief, LS-174T tumor-bearing female athymic mice were injected i.v. with approximately 100 μCi of ^86^Y-CHX-A"-DTPA-panitumumab F(ab')_2_. The reconstructed images were processed and analyzed using AMIDE (A Medical Image Data Examiner) software program. To minimize spillover effects, regions of interest (ROIs) were drawn to enclose approximately 80-90% of the organ of interest, avoiding the edges. To minimize partial-volume effects caused by non-uniform distribution of the radioactivity in the containing volume, smaller ROIs were consistently drawn to enclose the organ. Upon completion of the imaging session, the mice were euthanized and biodistribution studies were performed to determine the correlation between PET-assessed *in vivo *percent of injected dose per cubic centimeter and biodistribution-determined percent of injected dose per gram.

## Results

**Figure 1:**
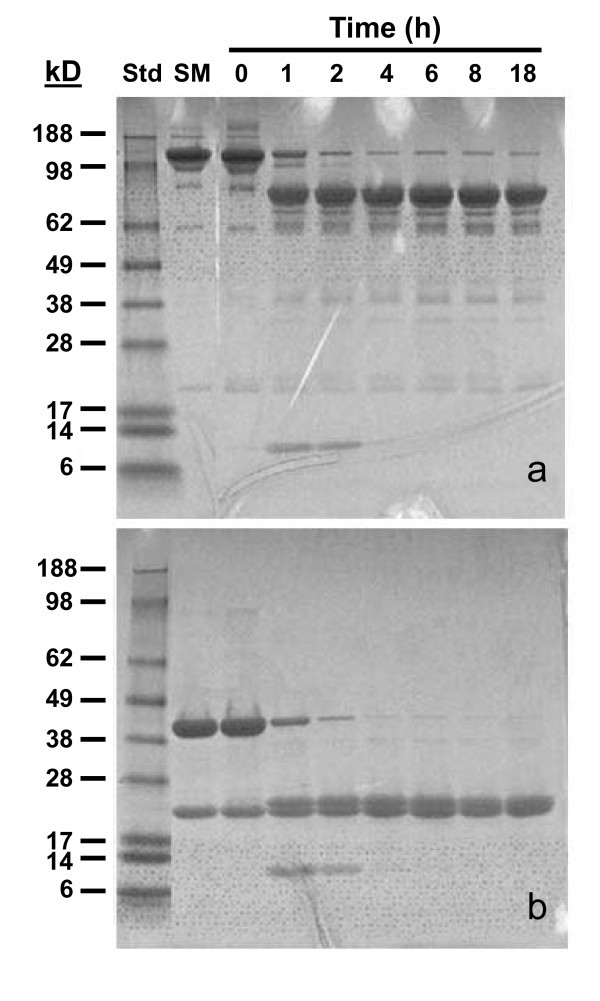
**SDS-PAGE analysis of peptic digest of panitumumab IgG**. Panitumumab was subjected to digestion with 2% pepsin at 37°C. At the specified time points, samples were neutralized and stored at 4°C until analyzed. The peptic digests were analyzed under non-reducing (**a**) and reducing (**b**) conditions.

The studies as performed were designed to evaluate the *in vitro *and *in vivo *properties of the panitumumab-CHX-A" F(ab')_2 _fragment and assess the potential of this molecule for imaging and therapeutic applications. To determine the optimal (digestion) cleavage time, 2% pepsin was added to 250 μg panitumumab and incubated at 37°C. Aliquots were removed at 1, 2, 4, 6, 8, and 18 h. As determined by SDS-PAGE, near complete pepsin digestion of panitumumab to a F(ab')_2 _fragment appears to occur after 8 h, evident in Figure 1 by the loss of the higher-molecular-weight band of the intact IgG under non-reducing conditions (Figure 1a) and the transition of the heavy-chain band to a lower molecular weight when subjected to reduction with β-mercaptoethanol (Figure 1b).

**Figure 2:**
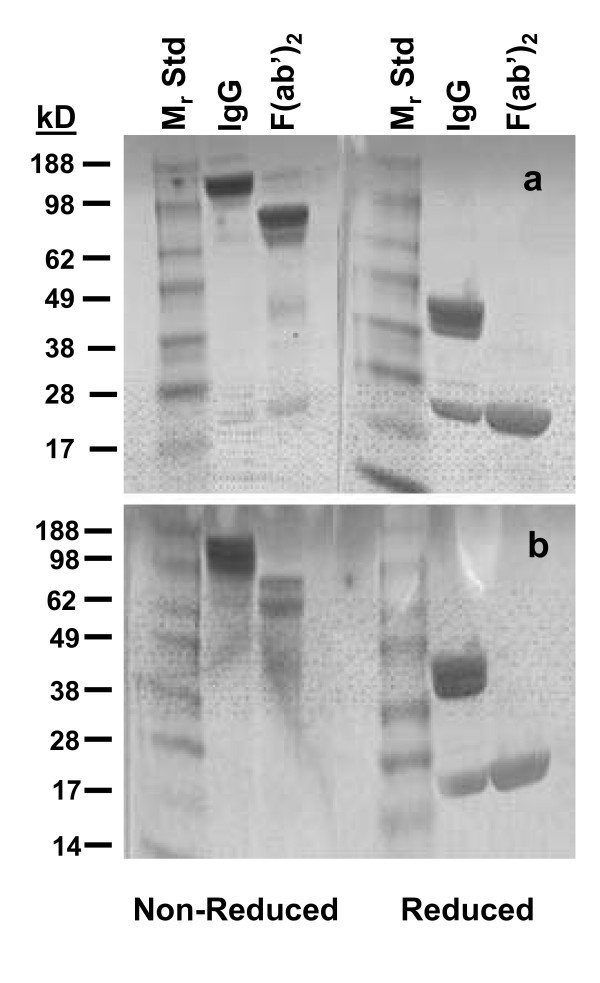
**SDS-PAGE analysis of panitumumab F(ab')_2_**. The panitumumab F(ab')_2 _was evaluated by SDS-PAGE before (**a**) and after (**b**) the final step of buffer exchange and concentration using a Centriprep centrifugal filtration device. The fragment was applied to a 4-20% gel in the absence and presence of β-mercaptoethanol.

Having determined the incubation time for the peptic digestion, 100 mg of panitumumab was digested overnight with 2% pepsin. When analyzed by SDS-PAGE, as expected, major bands were visualized corresponding to a molecular weight (M_r_) of 79.4 under non-reducing conditions while two bands were evident at 25.1 and 22.4 kD after reduction (Figure 2a). Lower-molecular-weight (LMW) species at approximately 38 and 22 kD were also evident under non-reducing conditions. These LMW species, with a retention time of 24.2 min on SE-HPLC, comprised 30.5% of the reaction mixture, most likely representing pepsin and the Fc fragment (data not shown). The retention time of the panitumumab F(ab')_2 _was 36 min by SE-HPLC, consistent with a M_r _of 89.1 kD using tandem TSK2000 and 4000 columns for the analysis. Following buffer exchange to phosphate-buffered saline and subsequent concentration of the F(ab')_2 _preparation using an Amicon Centriprep with a MWCO of 50 kD, the final product was again analyzed by SDS-PAGE and SE-HPLC: the LMW was no longer present as shown in Figure 2b. A final yield of 37.3 mg of panitumumab F(ab')_2 _was obtained as determined by protein quantitation by the Lowry method.

**Figure 3:**
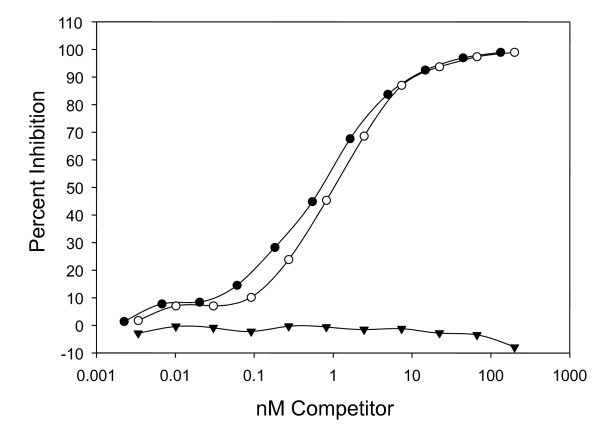
**Evaluation of panitumumab F(ab')_2 _immunoreactivity in a competition radioimmunoassay**. The immunoreactivity of panitumumab F(ab')_2 _(white circle) for purified EGFR was compared to panitumumab IgG (filled circle). The F(ab')_2 _of the anti-HER2 mAb, trastuzumab, (downward-pointing filled triangle) was used as a negative control.

The peptic digest appears to have a modest effect on the immunoreactivity of the panitumumab F(ab')_2 _fragment. When analyzed in a competition radioimmunoassay, depicted in Figure 3, the concentration for 50% inhibition (IC_50_) for intact panitumumab IgG was 0.5 nM while the IC_50 _for the panitumumab F(ab')_2 _was 1 nM.

Evaluating the potential of the panitumumab F(ab')_2 _for clinical imaging and radioimmunotherapy applications would require larger quantities of the F(ab')_2_. Therefore, a peptic digestion was performed with 1 g of panitumumab. As with the previous preparation, LMW species were detected by SE-HPLC which comprised approximately 34% of the digest mixture. For this larger preparation, a tangential flow filtration system with a 50 kD MWCO was used to eliminate the LMW species, exchange the buffer, and to also concentrate the F(ab')_2_. The final product, analyzed by SDS-PAGE and SE-HPLC, was found to be comprised of a single product consistent with the Mr of a F(ab')_2 _(data not shown). The final yield of this preparation was appreciably higher than the digestion of 100 mg with a final yield of 56%.

A trial conjugation of the panitumumab F(ab')_2 _with the acyclic ligand, CHX-A"-DTPA was then performed at a molar excess of 5:1, 10:1, and 20:1. These reactions resulted, respectively, in an average chelate to protein ratio of 2.9, 1.7, and 5.6 (Table 1). The immunoconjugates were evaluated in a competition radioimmunoassay to determine if the modification affected the immunoreactivity of the panitumumab F(ab')_2 _fragment. The modification with the CHX-A"-DTPA chelate had minimal effect on the immunoreactivity of the panitumumab F(ab')_2 _(Table 1). The IC_50 _for the 2.9, 1.7, and 5.6 was 0.6, 0.7, and 0.7 nM, respectively, compared to the unmodified panitumumab F(ab')_2 _IC_50 _of 0.5 nM.

**Table 1:** *In vitro *analysis of panitumumab F(ab')_2 _conjugated with CHX-A"-DTPA.

	Chelate:mAb ratio (molar excess)				
	Panitumumab F(ab')_2_				HuM195 F(ab')_2_
Analysis	0	5×	10×	20×	10×
IC_50 _(nM)	0.5	0.6	0.7	0.7	0
Specific activity (mCi/mg)	-	1.9	14.8	2.9	9.4
Labeling yield (%)	-	13.9	49.4	24.4	50
Chelate:protein ratio	-	2.9	1.7	5.6	0.6
Percent binding	-	59.5	59.4	54.7	1.8

Each of the immunoconjugate preparations were radiolabeled with ^111^In and their characteristics compared. The specific activities ranged from 1.9 to 14.8 μCi/μg, with the labeling efficiency ranging from 13.9% to 49.4% (Table 1). When the radiolabeled panitumumab F(ab')_2 _fragments were incubated with purified hEGFR for 4 h at 37°C, 54.7% to 59.5% of the radioactivity was bound. The addition of 10 μg of unlabeled fragment, which reduced the percentage of bound radioactivity to an average of 3.5%, demonstrated the specificity of the RICS. Specificity of the radioimmunoassay was also demonstrated with the lack of binding (1.8%) with ^111^In- HuM195 F(ab')_2_. Based on these data, the preparation with 1.7 chelates, from the 10:1 molar excess reaction, was chosen for the remaining studies. In subsequent assays, specific binding of the RIC with hEGFR was as high as 72%.

Biodistribution studies were performed to quantitate tumor targeting and to determine the normal organ distribution of the ^111^In- CHX-A"-panitumumab F(ab')_2 _fragment. Athymic mice bearing LS-174T xenografts were injected (i.v.) with ^111^In- -panitumumab F(ab')_2 _(approximately 7.5 μCi), the results are presented in Table 2. At 24 h, the percentage of injected dose per gram of the ^111^In-panitumumab F(ab')_2 _in tumor was 21.42 ± 7.67 and remained at this level for 72 h at which time the percentage of injected dose per gram was 21.55 ± 6.22. The percentage of injected dose per gram then decreased to 8.01 ± 3.65 by 168 h. Of the normal organs, the highest percentage of injected dose per gram (13.13 ± 2.34) was observed in the kidney at 24 h which decreased to 2.66 ± 0.46 by 168 h. The next highest normal organ uptake was observed in the liver with a percentage of injected dose per gram of 8.01 ± 1.63 at 24 h that decreased to 3.38 ± 0.67 by 168 h. The blood percentage of injected dose per gram was 6.84 ± 2.30 at 24 h, but then steadily decreased to 0.08 ± 0.02 by the end of the study (168 h). All other organs began with their highest percentage of injected dose per gram at 24 h and steadily decreased to the end of the study.

**Table 2:** Tumor targeting and normal organ distribution of i

	Time points (h)				
Tissue	24	48	72	96	168
Blood	6.84 ± 2.30	2.28 ± 0.53	1.12 ± 0.27	0.32 ± 0.12	0.08 ± 0.02
Tumor	21.42 ± 7.67	21.12 ± 2.85	21.55 ± 6.2	16.55 ± 2.35	8.01 ± 3.65
Liver	8.01 ± 1.63	4.98 ± 0.98	4.66 ± 0.62	3.55 ± 0.62	3.38 ± 0.67
Spleen	5.43 ± 1.64	3.61 ± 0.87	3.96 ± 1.19	2.63 ± 1.12	2.09 ± 0.80
Kidneys	13.13 ± 2.34	8.27 ± 0.84	6.00 ± 1.57	5.22 ± 0.94	2.66 ± 0.46
Lung	4.40 ± 1.14	2.23 ± 0.39	1.79 ± 0.31	1.06 ± 0.21	0.8 ± 0.26
Heart	3.57 ± 1.11	2.06 ± 0.23	1.86 ± 0.30	1.20 ± 0.21	0.78 ± 0.12
Femur	2.41 ± 0.48	1.78 ± 0.41	1.67 ± 0.24	1.01 ± 0.24	0.75 ± 0.22

Athymic mice bearing s.c. LS-174T xenografts were injected with ^111^In- CHX-A"-panitumumab F(ab')_2 _(approximately 7.5 μCi). At the indicated time points, the mice (*n *= 5) were sacrificed by exsanguination; the tumor, blood, and major normal organs were harvested, wet-weighed, and the radioactivity measured. The values represent the average percent injected dose per gram (%ID/g) of tissue along and the standard deviations.

The blood pharmacokinetics of ^111^In-panitumumab F(ab')_2 _was evaluated in tumor- and non-tumor-bearing mice following i.v. and i.p. administration. In the absence of a tumor burden, i.v. injected RIC demonstrated a clearance from the blood compartment that was nearly twofold slower, for the both T_1/2_α- and T_1/2_β phase, than what was obtained in mice bearing s.c. LS-174T xenografts (Table 3). When non-tumor bearing mice were injected with the RIC by an i.p. route, the T_1/2_β phase was similar to what was obtained for the i.v. injected route; 18.6 h for the i.v. injected group and 19.3 h for the i.p.-injected group. In contrast to the i.v.-injected sets of mice, the clearance (T_1/2_β phase) of the RIC following i.p. injection in the mice bearing i.p. tumor xenografts was only 5.6 h longer than what was obtained in the mice that were tumor free.

**Table 3:** Biphasic analysis of blood pharmacokinetics of ^111^In- CHX-A"-panitumumab following i

Tumor site	Injection route	Blood clearance		
		α^a ^(h)	β (h)	***r***^**2**^
None	i.v.	1.2	18.6	0.996
s.c.	i.v.	0.6	10.4	0.998
None	i.p.	-	19.3	0.98
i.p.	i.p.	-	24.9	0.917

^a^Non-tumor bearing mice or mice bearing s.c. or i.p. LS-174T xenografts were injected by intravenous or intraperitoneal injection with approximately 7.5 μCi of ^111^In- CHX-A"-panitumumab F(ab')_2_. Blood samples (10 μL) were drawn over a 1-week period and measured in a γ-counter. The percentage of injected dose per milliliter plotted and the T_1/2_α- and T_1/2_β-phase values calculated using SigmaPlot 9.

**Figure 4:**
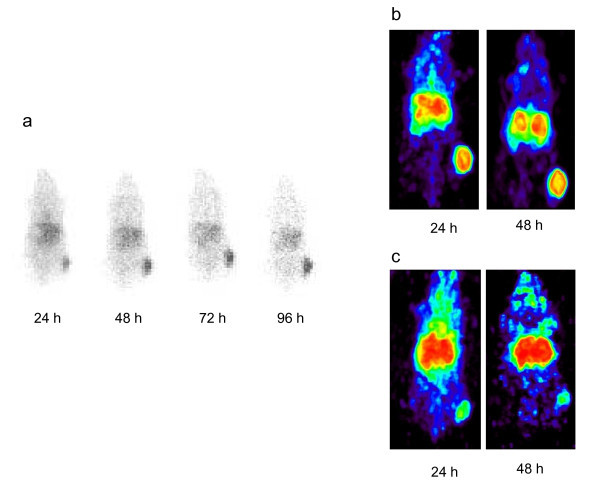
**Validation of panitumumab F(ab')_2 _as an imaging agent of HER1 positive tumors**. (**a**) γ-Scintigraphy was performed with mice bearing LS-174T s.c. tumor xenografts. Following i.v. injection with approximately 100 μCi of ^111^In-CHX-A"-panitumumab F(ab')_2_, mice were imaged over a 4-d period. Positron-emission tomograhic (PET) using ^86^Y-CHX-A"-panitumumab F(ab')_2_. (**b**) Mice bearing s.c. LS-174T xenografts were injected i.v. with (50-60 μCi) of ^86^Y-CHX-A"-panitumumab F(ab')_2 _and imaged 1 and 2 days post-injection of the RIC. (**c**) Specificity was demonstrated by co-injecting 100 μg of panitumumab with the RIC and blocking uptake of the RIC by the tumor.

Tumor targeting of the panitumumab F(ab')_2 _was also validated through the use of two imaging modalities, planar γ-scintigraphy, and positron-emission tomography (PET). The s.c. LS-174T tumors on the rear flank of the mice were clearly visualized by planar γ-scintigraphy (Figure 4a). Over the 4-day period that images were collected, not only does the ^111^In-labeled panitumumab remain in the tumor, but the RIC clears from the body.

Imaging was also performed with ^86^Y-CHX-A"-DTPA-panitumumab F(ab')_2 _on the ATLAS. Mice bearing the LS-174T xenografts were injected i.v. with approximately 100 μCi (3 μg) of ^86^Y- panitumumab F(ab')_2_. Images were taken at 24 and 48 h; after the 48 h images were collected, the mice were euthanized and the tumor, blood, and normal organs were harvested to obtain direct counts to correlate with the quantitation by imaging. All of the tumors were clearly visualized for both days following injection of the RIC as shown in the maximum intensity image (Figure 4b). The blood pool (heart, lungs, liver) is visible in these images, but it appears to have decreased on the second day while the tumor uptake increased. Specificity is demonstrated by reduction in tumor uptake when 0.1 mg of unlabeled panitumumab was co-injected with the ^86^Y-labeled panitumumab F(ab')_2 _(Figure 4c).

**Figure 5:**
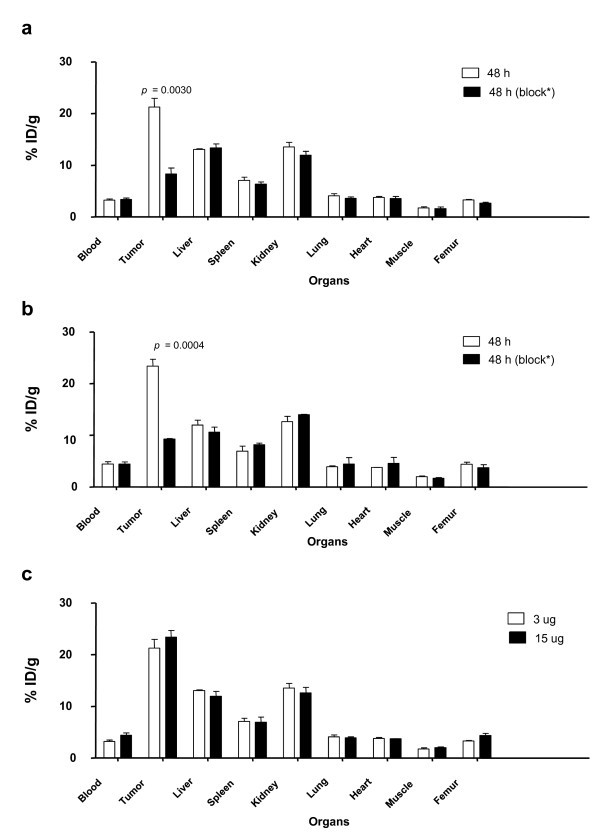
**Quantitation of tumor and normal organ distribution of ^86^Y-CHX-A'-panitumumab F(ab')_2_**. (**a**) Receptor-mediated uptake of ^86^Y-CHX-A"-panitumumab F(ab')_2 _of LS-174T tumor xenografts and normal organs 2 days post-injection of the RIC. Data represent the mean ± SEM from at least three determinations. (**b**) The specific activity of the ^86^Y- CHX-A"-panitumumab F(ab')_2 _was lowered by the addition of 15 μg of panitumumab F(ab')_2_. The mice were euthanized following the completion of the 48-h imaging session, the blood, tumor and normal organs were harvested and the radioactivity measured. (**c**) Comparison of the 48 h *ex vivo *quantitation at the two concentrations of panitumumab F(ab')_2_.

Direct quantitation of the distribution of ^86^Y-panitumumab F(ab')_2 _fragment in the liver and tumor provided results similar to what was obtained with the ^111^In-labeled fragment (Figure 5a). The percentage of injected dose per cubic centimeter calculations from the images correlated (Table 4) with the *ex vivo *percentage of injected dose per gram quantitation (*r*^2 ^= 0.91, *p *= 0.89). Finally, when the specific activity of the ^86^Y-panitumumab F(ab')_2 _was lowered by the addition of 15 μg of panitumumab F(ab')_2 _(Figure 5b); no mass effect was observed in the level of radioactivity in the blood, tumor, or normal organs as determined by imaging and *ex vivo *quantitation (Figure 5c and Table 4).

**Table 4:** Quantitation of tumor and liver uptake of ^86^Y-CHX-A"-panitumumab F(ab')_2 _by PET imaging.

			Time post-injection (h)	
Protein dose	Blocked	Organ	24	48
3 μg	No	Tumor	20.31 ± 1.52^a^	22.59 ± 1.51
		Liver	12.98 ± 0.95	11.68 ± 0.43
				
	Yes	Tumor	11.37 ± 0.58	9.92 ± 0.66
		Liver	12.34 ± 0.99	12.95 ± 0.88
				
15 μg	No	Tumor	20.30 ± 1.01	22.98 ± 0.84
		Liver	13.84 ± 0.5	11.62 ± 1.12
				
	Yes	Tumor	11.44 ± 0.69	11.38 ± 0.96
		Liver	12.60 ± 0.70	10.66 ± 0.57

The values presented are the percentage of injected dose per cubic centimeter. Receptor-mediated uptake of ^86^Y-CHX-A"-panitumumab F(ab')_2 _of LS-174T tumor xenografts and normal organs 2 days post-injection of the RIC. Data represent the mean ± SEM from at least three determinations. The specific activity of the ^86^Y- CHX-A"-panitumumab F(ab')_2 _was lowered by the addition of 15 μg of panitumumab F(ab')_2_.

**Figure 6:**
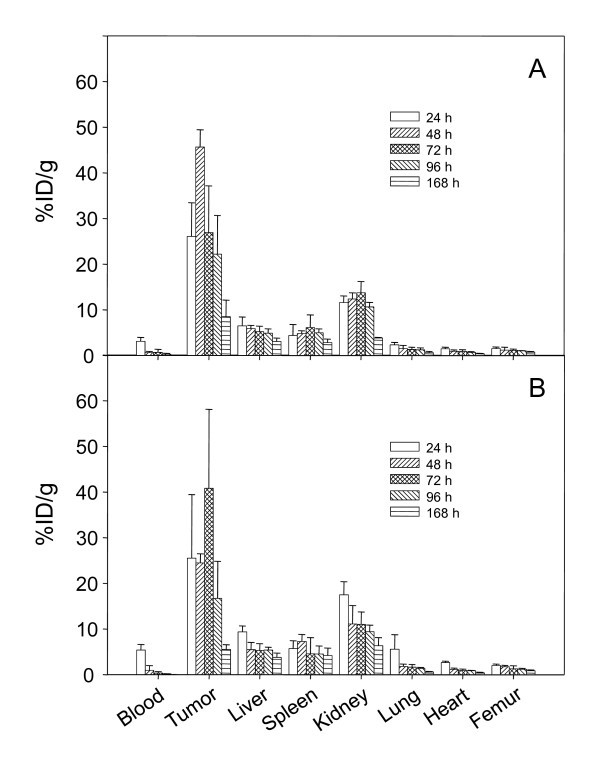
**Tumor and normal organ distribution of ^111^In-CHX-A"-panitumumab F(ab')_2 _in mice (*n *= 5) bearing i.p. LS-174T tumor xenografts were injected i.v. (a) or i.p. (b) with ^111^In-CHX-A"-panitumumab F(ab')_2 _(approximately 7.5 μCi)**. Mice were euthanized at the indicated times, blood, tumor, and organs were harvested, wet-weighed, and the radioactivity measured. The percentage of injected dose per gram with the standard deviations is plotted.

As a prelude to: (1) utilizing the panitumumab F(ab')_2 _for monitoring response to radioimmunotherapy and (2) exploiting the panitumumab F(ab')_2 _as a targeting vector for radioimmunotherapy of disseminated peritoneal disease, direct quantitation of intraperitoneal (i.p.) tumor xenograft targeting was performed. The targeting of i.p. tumors by ^111^In-panitumumab F(ab')_2 _was evaluated using both an i.p. and i.v. injection route, the results of which are presented in Figure 6. Not unexpectedly, i.p. administration of the RIC resulted in excellent targeting of the i.p. tumors (Figure 6a). Peaking at 48 h, the tumor percentage of injected dose per gram was 45.67 ± 3.79 and declined to 8.50 ± 3.63 at 168 h. When mice bearing i.p. tumor xenografts were given an i.v. injection of the RIC, the peak tumor percentage of injected dose per gram was at a similar level to the aforementioned experiment; however, this maximum did not occur until 72 h (Figure 6b). The pattern of normal organ distribution in these last two studies was similar to what was obtained with the i.v. injected ^111^In-labeled panitumumab F(ab')_2 _already discussed.

## Discussion

The *in vivo *and *in vitro *properties of the intact panitumumab mAb has been previously described by Ray et al. [25] which included imaging by planar γ-scintigraphy. The potential of imaging EGFR-positive tumors for purposes of monitoring disease response to therapy and performing dosimetric calculations was successfully extended to PET imaging with ^86^Y-panitumumab [26,36]. While the ^111^In-CHX-A"-panitumumab demonstrated tumor uptake in LS-174T xenografts [25], maximal localization of the intact mAb does not occur until 48-96 h post-injection [13]. The objective of this study was to evaluate the *in vivo *and *in vitro *properties of panitumumab F(ab')_2 _fragment and to assess its utility as a targeting agent in radioimmunodiagnostic and radioimmuntherapeutic protocols. To date, this report appears to be the first such characterization of a fragment generated from panitumumab.

Although intact monoclonal antibodies have been considered candidates for targeted therapy due to their specificity, with their long residence time in the blood of days to weeks, they are not ideal carriers for imaging probes; it is extremely difficult to perform serial imaging of less than several days [37,38]. Early studies demonstrated that the size of antibody-based imaging agents are inversely related to their blood clearance; the clearance rate of Fab or Fab' > F(ab')_2 _> IgG [13,15]. Covell et al. [15] found that the whole murine IgG was retained in the (mouse) body 17 times longer than F(ab')_2_. Consequently, normal tissue exposure is much greater with the intact antibody than it is for the fragment.

The Fab fragments, smaller in size, clear even faster than an F(ab')_2 _fragment and have been developed predominantly as imaging agents [13,15,39]. Their limitations pertain to the fact that they are monovalent and their molecular weight subjects them to efficient glomerular filtration. Monovalency often results in the loss of functional affinity and reduces the binding strength of the Fab or Fab' fragment as compared to the F(ab')_2 _or IgG [39,40]. In 1983, Wahl and colleagues reported on a direct comparison of three radio-iodinated mAb forms, anti-CEA IgG, F(ab')_2 _and Fab using γ-scintigraphy. Interestingly, the F(ab')_2 _exhibited the fastest tumor localization [14]. At 2 days post-injection of the ^131^I-anti-CEA-F(ab')_2 _fragment tumor was clearly visualized. By the third day the images were equivalent to those obtained at 11 days with the intact mAb. The authors concluded that Fab fragments were not an optimal vector for imaging due to their rapid clearance, low accumulation in tumor and high renal accumulation. Similar observations have been noted with other mAbs. As reviewed by Tolmachev [40], ^111^In-DTPA-trastuzumab-Fab tumor uptake as compared to that of ^111^In-DTPA-trastuzumab-F(ab')_2 _is considerably lower. In contrast, an early clinical imaging study conducted by Delaloye et al. [19] compared ^123^I-labeled Fab and F(ab')_2 _fragments of an anti-carcinomembryonic antigen mAb in colorectal carcinoma patients for the detection of disease using emission-computed tomography. The Fab fragment was reported to have clearer images than those of the F(ab')_2 _fragment with a higher overall detection of tumor lesions. The authors postulate that the success of these studies was the result of careful selection and matching of the target, the targeting vehicle, and the radionuclide.

Based on these earlier reports and the importance of retaining affinity/avidity, the F(ab')_2 _fragment was selected for this investigation as the targeting vehicle to be exploited in imaging modalities, e.g., PET, planar γ-scintigraphy, MRI, and optical. In this study, F(ab')_2 _fragments were successfully generated from panitumumab by peptic digest and the protocol developed was readily scaled-up. The *in vitro *analysis, SDS-PAGE and SE-HPLC, indicated that the final product has a Mr of 79.4 and 89.1 kD, respectively, and retained immunoreactivity for HER1. Once the F(ab')_2 _fragment was generated, conjugation with the bifunctional chelate, acyclic CHX-A"-DTPA was performed for radiolabeling with medically relevant radionuclides such as ^111^In and ^86^Y which is also appropriate for radiolabeling with therapeutic radionuclides such as ^177^Lu, ^213^Bi, and ^212^Bi [41-43]. The conjugation was performed at three different molar ratios of chelate to panitumumab F(ab')_2_. The different ratios had minimal effect on the immunoreactivity of the panitumumab F(ab')_2 _as demonstrated by a competition radioimmunoassay. Furthermore, the specific binding of the immunoconjugate preparations with hEGFR was comparable following radiolabeling with ^111^In. Based on the results, the 10:1 molar excess ratio (1.7 chelate/antibody) was chosen for the remaining studies.

This represents the first report on the use of a panitumumab fragment for imaging applications. As such, comparisons to other mAb fragments will have to suffice in the discussion of the data reported herein. Smith-Jones (2004) and co-workers conjugated the macrocyclic ligand DOTA to trastuzumab F(ab')_2_. reporting an average of 6.3 chelates per F(ab')_2 _fragment and immunoreactivity of 81% [37]. No other *in vitro *assay data was reported, i.e., assessing the effect of the fragmentation of the mAb, or the chelate number, on the overall immunoreactivity of the mAb. A number of studies have investigated the use of F(ab')_2 _fragments with radio-iodines for imaging and radioimmunotherapy, making a direct comparison with a metallic radionuclide difficult [12,18,19,44,45]. For example, an F(ab')_2 _of the mAb14C5 was evaluated for the radioimmunodetection of non-small cell lung tumor xenografts [45]. Some targeting was observed; however, tumors were poorly visualized by γ-scintigraphy. More promising results were reported for the ^125^I-labeled F(ab')_2 _fragment of mAb B6.2 [12]. In this case, excellent tumor targeting was demonstrated by direct quantitation studies as well as γ-scintigraphy.

In contrast to panitumumab IgG, the F(ab')_2 _attains a higher percentage of injected dose per gram in the LS-174T s.c. tumor (21.42 ± 7.67) at an earlier time point (24 h) than the intact IgG (13.27 ± 8.40) following i.v. administration [25]. The F(ab')_2 _fragment may not achieve the same level of targeting, it is, however, retained in the tumor. In contrast to the intact IgG, the F(ab')_2 _is rapidly cleared from the blood compartment, evident by the biodistribution and pharmacokinetic data presented. At 24 h, the blood percentage of injected dose per gram of panitumumab F(ab')_2 _is approximately one-half of that reported for panitumumab IgG. The differential becomes even greater by 7 days. The blood pharmacokinetics of the panitumumab F(ab')_2 _in tumor-bearing mice reported here is consistent with that of other F(ab')_2 _fragments described in the literature [13]. Interestingly, when compared to panitumumab IgG, there is a "reversal" of the values for the T_1/2_α and β values of the tumor and non-tumor bearing mice. In the absence of a tumor burden, the T_1/2_α and β for IgG was approximately 5.2 and 3.8 times faster, respectively, than in the presence of tumor. In contrast, the T_1/2_α and β of the blood clearance for the ^111^In-panitumumab F(ab')_2 _was approximately two times faster in the tumor-bearing mice vs. in mice without a tumor burden. One explanation for this phenomenon is that murine FcRn cross-reacts with and will bind human IgG which results in prolonging its half-life in the mouse [38,46]. The F(ab')_2_, lacking the Fc region, is likely cleared by phagocytic cells of the reticuloendothelial system.

The results reported herein are comparable to studies performed with F(ab')_2 _of other mAbs. In a study with ^111^In-DOTA-Herceptin F(ab')_2_, the tumor percentage of injected dose per gram was 20.4 ± 6.8 after 24 h. At the same time point, a tumor percentage of injected dose per gram of 21.42 ± 7.67 was obtained with ^111^In-CHX-A"-DTPA-panitumumab F(ab')_2_. Differences between the two studies are evident in renal uptake of the RICs. The kidney percentage of injected dose per gram of uptake ^111^In-CHX-A"-DTPA-panitumumab F(ab')_2 _was only 13.13% at 24 h compared to approximately 65% with ^111^In-DOTA -Herceptin F(ab')_2 _[37]. A F(ab')_2 _fragment of the recombinant anti-L1 CAM mAb, chCE7, has also been evaluated for PET imaging when radiolabeled with ^64^Cu and for radioimmunotherapy using ^177^Lu [47]. Maximal tumor uptake of the ^177^Lu-labeled RIC was observed at 24 h with an ID/g of 14.43 while maximum uptake for the ^67/64^Cu-labeled RIC occurred at 8 h. Unfortunately, kidney uptake with either of these radiolabeled chCE7 F(ab')_2 _preparations was greater than what was observed in the tumor at all time points of the study.

The panitumumab F(ab')_2 _also appears to be a potential vehicle for targeting disseminated intraperitoneal disease. Whether administered i.v. or i.p., excellent tumor targeting of the i.p. tumor xenografts was observed, with a tumor percentage of injected dose per gram as high as 45.66, 2.1-fold greater than the highest value calculated for the s.c. tumor following i.v. administration. Interestingly, the peak tumor percentage of injected dose per gram of both the s.c. and i.p. tumors following i.v. injection occurred at 72 h whereas the peak i.p. tumor value occurred at 48 h following i.p. injection of the panitumumab F(ab')_2_. Imaging studies, γ-scinitigraphy and PET, of the i.p. tumor model using these two injection routes are pending.

Studies from this laboratory and others have demonstrated the potential of panitumumab for the non-invasive monitoring of HER1-positive tumors and quantitating HER1 expression using modalities such as γ-scintigraphy, PET, and optical imaging [24,25,36,48,49]. By evidence of the tumor targeting, direct quantitation of tumor targeting suggests that the ^111^In-labeled panitumumab F(ab')_2 _is stable *in vivo*. This observation is also supported by the imaging studies related herein. This attribute may be due in part to the fact that panitumumab is of the IgG_2 _immunoglobulin subclass. In a series of dual-labeled experiments, Buchegger et al. [44] compared F(ab')_2 _fragments generated from chimeric mAb of three subclasses (IgG_1_, IgG_2_, and IgG_4_) directed against carcinoembryonic antigen. The *in vivo *studies revealed that the IgG_2 _F(ab')_2 _fragment had superior tumor targeting and possessed the longest biological half-life as well. The poorest results were obtained with the F(ab')_2 _of the IgG_4_. The radio-iodinated F(ab')_2 _fragment of chimeric 81C6, another IgG_2 _subclass mAb, has also been found stable and suitable for imaging applications [18].

The images presented here, both planar γ-scintigraphy and PET, indicate that there is excellent uptake of the panitumumab F(ab')_2 _by 24 h with decreasing background over the course of the imaging sessions, consistent with the studies conducted by Wahl and colleagues [14]. The *ex vivo *quantitation of tumor and tissue uptake (percentage of injected dose per gram) correlated with the quantitation (percentage of injected dose per cubic centimeter) performed from the PET images. These data were comparable to those obtained with panitumumab IgG, providing confidence in the suggestion that the panitumumab F(ab')_2 _would be useful in assessing tumor responses to therapy (i.e., estimating HER1 expression) and would provide accurate data for performing dosimetric calculations for radioimmunotherapy [36]. The caveat to translating the imaging of HER1 with panitumumab, either the intact IgG or a fragment, is whether or not human hepatocytes will be targeted. Clinical studies using ^111^In- or ^99m^Tc-labeled cetuximab reported visualization of tumor burden in patients with squamous-cell carcinoma of the lung, head, and neck with significant hepatic uptake of radioactivity [50,51]. Panitumumab may prove superior in this aspect. In pre-clinical studies, cetuximab has been observed to have a higher hepatic uptake of radioactivity than panitumumab [25,29].

Alternative to enzymatically generated fragments of monoclonal antibodies are a variety of genetically engineered forms (i.e., sfv, minibody, diabody, domain-deleted) that are under evaluation for imaging and therapeutic applications. The valency and molecular weight of these mAb forms have been tailored to alter such biological properties as their blood residence time, tumor penetration, tumor residence time, renal clearance, and catabolic susceptibility [11,39,52-54]. Renal and hepatic uptake of many of these forms remains an obstacle when labeled with a metallic radionuclide limiting their potential for imaging to the radio-iodines or ^18^F.

This study demonstrates that F(ab')_2 _fragments of the anti-EGFR mAb panitumumab can be generated efficiently and quickly. The immunoreactivity of the panitumumab F(ab')_2 _fragment is comparable to the IgG. The immunoreactivity is retained on further modification with the chelating agent, acyclic CHX-A"-DTPA and subsequent radiolabeling. The study also demonstrated tumor targeting with LS-174T xenografts. Because of the bivalency, fast blood clearance, and deep tumor penetration, the panitumumab F(ab')_2 _fragment is a good candidate for imaging. Studies are continuing with the panitumumab F(ab')_2 _to further evaluate the role of panitumumab F(ab')_2 _in radiological imaging, SPECT and PET, and also for its potential role in targeted MRI.

## Competing interests

The authors declare that they have no competing interests.

## Authors' contributions

KJW performed the *in vitro *and *in vivo *studies. KEB and KG carried out radiolabelings. TN performed PET imaging and analysis. MWB synthesized the chelate and participated in the design of the studies. DEM conceived of and coordinated the studies and assisted with the *in vivo *experiments. All authors assisted in the drafting of the manuscript, the final version of which has been read and approved by all of the authors.
